# MicroRNA-217 attenuates intima–media complex thickness of ascending aorta measured by ultrasound bio-microscopy and inhibits inflammation and lipid metabolism in atherosclerotic models of ApoE^−/−^ mice

**DOI:** 10.1186/s12944-018-0825-2

**Published:** 2018-07-24

**Authors:** Haina Liu, Xia Li, Yanpeng Song, Zhibin Wang

**Affiliations:** 1 Department of Ultrasonography, The Affiliated Qingdao Municipal Hospital of Qingdao University, No.1 Jiaozhou Road, Qingdao, 266011 China; 2 Department of Ultrasonography, Jiaozhou Central Hospital of Qingdao, No.29 Xuzhou Road, Jiaozhou, 266300 China; 3 Department of Ultrasonography, The Affiliated Hospital of Qingdao University, No.16 Jiangsu Road, Qingdao, 266003 China

**Keywords:** MicroRNA-217, Atherosclerosis, Ultrasound bio-microscopy, Inflammation, Lipid

## Abstract

**Background:**

Little investigation was done to test the efficiency of microRNA-217 (miR-217) on atherosclerosis in vivo.

**Methods:**

ApoE^−/−^ mice were used to construct atherosclerotic models and ultrasound bio-microscopy (UBM) was applied to detect the intima–media thickness (IMT) of the ascending aorta. The serum level of miR-217 and correlation with IMT was investigated. After miR-217 mimic administration, the IMT, inflammation, and lipid-associated molecules were assayed.

**Results:**

The serum level of miR-217 was reduced in ApoE^−/−^ mice and showed a negative correlation with the IMT of the ascending aorta (r^2^ = 0.5899, *p* < 0.0001). miR-217 mimic administration attenuated IMT and down-regulated the level of serum triglyceride (TG), total cholesterol (TC), and low-density-lipoprotein cholesterol (LDL-C), while it could up-regulate high-density lipoprotein cholesterol (HDL-C). Inflammation relevant genes, such as F4/80, tumor necrosis factor (TNF)-α, interleukin (IL)-1, IL-6, and monocyte chemoattractant protein (MCP)-1, and lipid metabolism associated gene, such as LDL receptor, class A scavenger receptors (SR-A), scavenger receptor class B type I (SR-BI), CD36, ATP binding cassette subfamily A member 1 (ABCA1), and ATP binding cassette subfamily G member 1 (ABCG1) in the aorta were significantly down-regulated in miR-217 group when compared with atherosclerosis group.

**Conclusion:**

miR-217 could down-regulate IMT and modulate the inflammation and lipid metabolism process, which indicates that miR-217 could be a potential treatment option.

## Background

Atherosclerosis (AS) and its relevant complications, such as stroke and myocardial infarction, are the primary cause of morbidity and mortality globally. AS can be initiated by lipid deposit, further provoked by chronic inflammation, and ultimately processed into vulnerable foams to cause thrombosis or stenosis with potential distal ischemia [1–3]. Plaques lesions develop in the intima (the inner lining of the arteries) and progressively affect the media and the adventitia, so the intima–media complex thickness (IMT) can reflect the size and severity of plaques. It is also reported that the rate of plaques formation is aggressive in high-risk atherosclerosis patients, who have hypertension, smoking, obesity, and diabetes mellitus [4].

It is worth noting that early stage plaques can spontaneously regress and intermediate and advanced stages plaques show continuously and substantively progressive [5]. More understanding of the potential steps of atherosclerosis development and the mechanism related to plaques formation will give great help to clinical practice [6].

More experimental evidence has confirmed the key roles of microRNAs in balancing the pro- and anti-inflammatory cytokines expression [7, 8], regulating endothelial cell (EC) senescence [9–11], and adjusting vascular smooth muscle cells (VSMCs) proliferation and migration involved in atherosclerosis [12–14]. MicroRNA-217 (miR-217) has also been testified to regulate VSMCs proliferation and migration [15] and promotes endothelial senescence [10]. While little research is focused on the association of miR-217 and plaque initiation, progression, and rupture [16].

In this research, apolipoprotein E^−/−^ (apoE^−/−^) mice were used as an animal model of atherosclerosis to investigate the correlation of miR-217 with the IMT of ascending aorta and its role in inflammation and lipid metabolism process.

## Methods

### Mice

Apolipoprotein E (ApoE)^−/−^ mice (6–8 weeks old) were ordered from Peking Vital River Laboratory Animal Ltd. (Beijing, China) and fed with the high-fat diet (HFD, 20% fat, 20% sugar, and 1.25% cholesterol) for 3 months as previously reported to induce atherosclerosis [17]. The ApoE^−/−^ mice were further classified into three groups at random: 10 for the AS group, 10 for the miR-217 group, and 10 for the miRNA nonspecific control (NS-miR group). Ten wild-type C57BL/6 J mice served as a normal control. The miR-217 group and NS-miR groups were injected with miRNA-217 mimic (5’-TACTGCATCAGGAACTGATTG-3′) or corresponding control via tail vein at a dose of 80 mg /kg (200 μl saline, once a week for 4 weeks). The AS group and control group were administrated with 200 μl saline once a week for 4 weeks. All experimental protocols were approved by the Ethics Committee of The Affiliated Qingdao Municipal Hospital of Qingdao University. All animal maintenances were performed in accordance with the eighth edition of the National Institutes of Health Guide for the Care and Use of Laboratory Animals (2011) [18].

### The intima-media complex thickness (IMT) measurement

Ultrasound bio-microscopy (UBM) was applied to measure the intima-media complex thickness (IMT) using Vevo® 770 (Visual-Sonics Inc.) in accordance with validated protocols in human beings. The distance from the leading edge of the lumen–intima interface to the media–adventitia interface was defined as IMT. Before measurements, mice were anesthetized with 0.5% pentobarbital sodium (45 mg/kg, intraperitoneal injection, resolved in 200 μl phosphate-buffered saline solution) to yield 300 to 400 heart beats/min. The aortic valve ring and the brachiocephalic artery branch were used as anatomic landmarks to fix the measurement site of the ascending aorta during long-axis and short-axis images studies to capture the largest cross-sectional vessel area. All measurements were performed at least 3 times at the same vascular site, and all images were analyzed by two operators.

### Quantitative real-time RT-PCR (qRT-PCR)

Total RNA was extracted from the serum and aorta with QIAGEN miRNeasy Mini Kit (Qiagen, Valencia, CA, USA) according to the manufacturer’s instructions. 1 μg of RNA was reverse transcribed using High-Capacity cDNA Reverse Transcription kits (Applied Biosystems, Foster City, CA) and the detection of the mRNA levels was performed with SYBR Green master mix (Roche, Mannheim, Germany). The reaction procedures were as follows: 95 °C for 10 min, 40 cycles of 95 °C for 15 s, and 60 °C for 1 min. Relative gene expression levels were quantified using the comparative ΔCT method and β-actin was used as the reference gene. Primer sequences were listed in Table 1.

**Table 1:** Primers used for transcript quantification by real-time PCR

Name	Sequence
MiR-217	FOR: ACACTCCAGCTGGGTACTGCATCAGGAAC
	REV: TGGTGTCGTGGAGTCG
β-actin	FOR: CTGGGACGACATGGAGAAAA
	REV: GACCAGGTTGCCCATCACT
F4/80	FOR: TCTGGGGAGCTTACGATGG
	REV: TAGGAATCCCGCAATGATGG
TNF-α	FOR: GCCACCACGCTCTTCTGTC
	REV: TGCTCCTCCACTTGGTGGTT
IL-1	FOR: TTGACGGACCCCAAAAGATG
	REV: AGCTGCCACAGCTTCTCCAC
IL-6	FOR: CCATCCAGTTGCCTTCTTGG
	REV: TGCAAGTGCATCATCGTTGT
MCP-1	FOR: AGGTCCCTGTCATGCTTCTG
	REV: TCTGGACCCATTCCTTCTTG
CD36	FOR: GAGCAACTGGTGGATGGTTT
	REV: GCAGAATCAAGGGAGAGCAC
LDL-R	FOR: CCCACATCTGCAAGGACCTC
	REV: CTATGGAGCCCACAGCCTTG
ABCA1	FOR: GAACAGCTCCAGCTCCTCCA
	REV: TCCACGTCTTCCTCGGTGTT
ABCG1	FOR: CCTCACCCAGTTCTGCATCC
	REV: AGGGCAGCAAACATGAGGAA
SR-A1	FOR: CCTTCATTCAAGGGCCTCCT
	REV: CTCCTGGGTTTCCTCGACCT
SR-B1	FOR: CAAGCAGCAGGTGCTCAAGA
	REV: GGTTCTCCACGAAGGACACG

### Measurements of serum lipids

Serum triglyceride (TG), total cholesterol (TC), and high-density lipoprotein cholesterol (HDL-C) were respectively measured with the Triglyceride E-test (Wako, Osaka, Japan), cholesterol E-test (Wako, Osaka, Japan) and HDL E-test (Wako, Osaka, Japan). Serum low-density-lipoprotein cholesterol (LDL-C) level was further calculated with the Friedewald equation: LDL-C = (TC) - (HDL-C) - (TG/5).

### Statistical analysis

Statistical analysis was performed using SPSS version 16.0 software (SPSS, Inc., Chicago, IL, USA). Data were showed as the mean ± standard deviation and analyzed by *t-*test. *p* < 0.05 was considered to be statistically significant.

## Results

### miR-217 expression correlates with the IMT of the ascending aorta

**Fig. 1:**
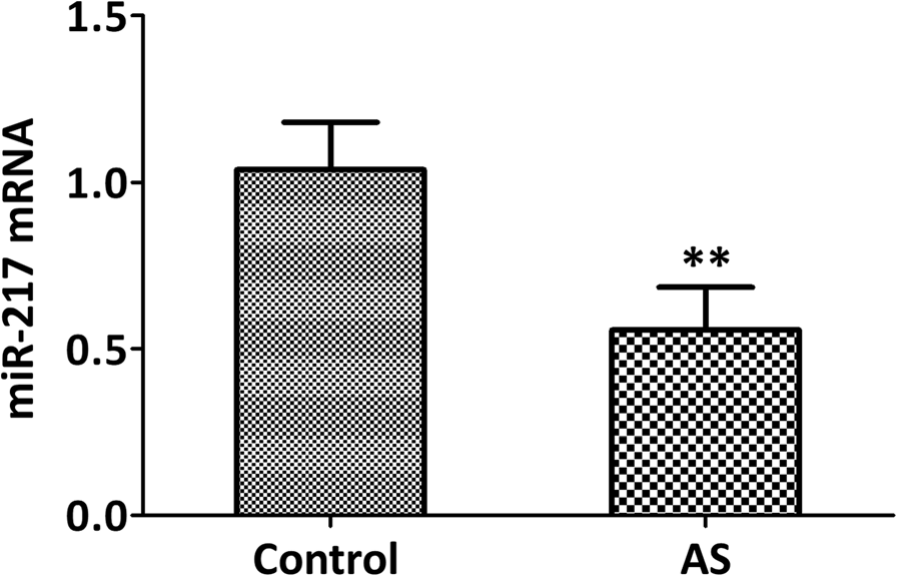
The level of miR-217 was reduced in the serum of atherosclerotic models of ApoE^−/−^ mice. qRT-PCR assay was applied to detect the expression levels of miR-217. Wild-type C57BL/6 J mice were used as control group. AS: atherosclerosis. *N* = 5 mice, **p < 0.01 vs. control group

**Fig. 2:**
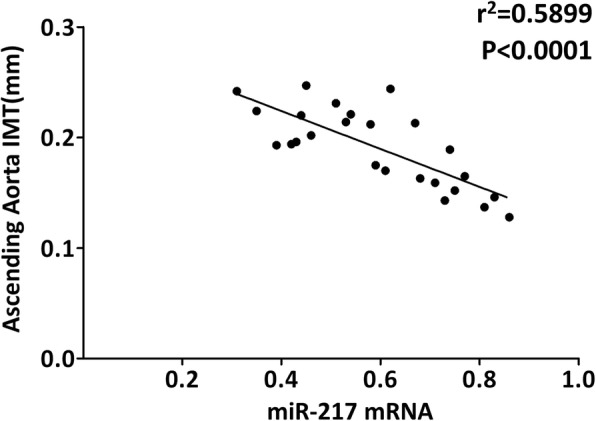
The level of miR-217 significantly correlated with intima–media thickness (mm) at the ascending aorta measured by ultrasound bio-microscopy (UBM) in atherosclerotic models of ApoE^−/−^ mice. *N* = 25 mice

The expression level of miR-217 in the serum of ApoE^−/−^ mice (atherosclerotic models) was significantly reduced compared with control group (Fig. 1, *p* < 0.01), which also showed a clear correlation with the IMT of the ascending aorta measured by UBM in ApoE^−/−^ mice (Fig. 2, *r*^2^ = 0.5899, *p* < 0.0001). All of these indicated that miR-217 was negatively correlated with the formation or size of plaque.

### miR-217 attenuates ascending aorta IMT and serum lipid levels

**Fig. 3:**
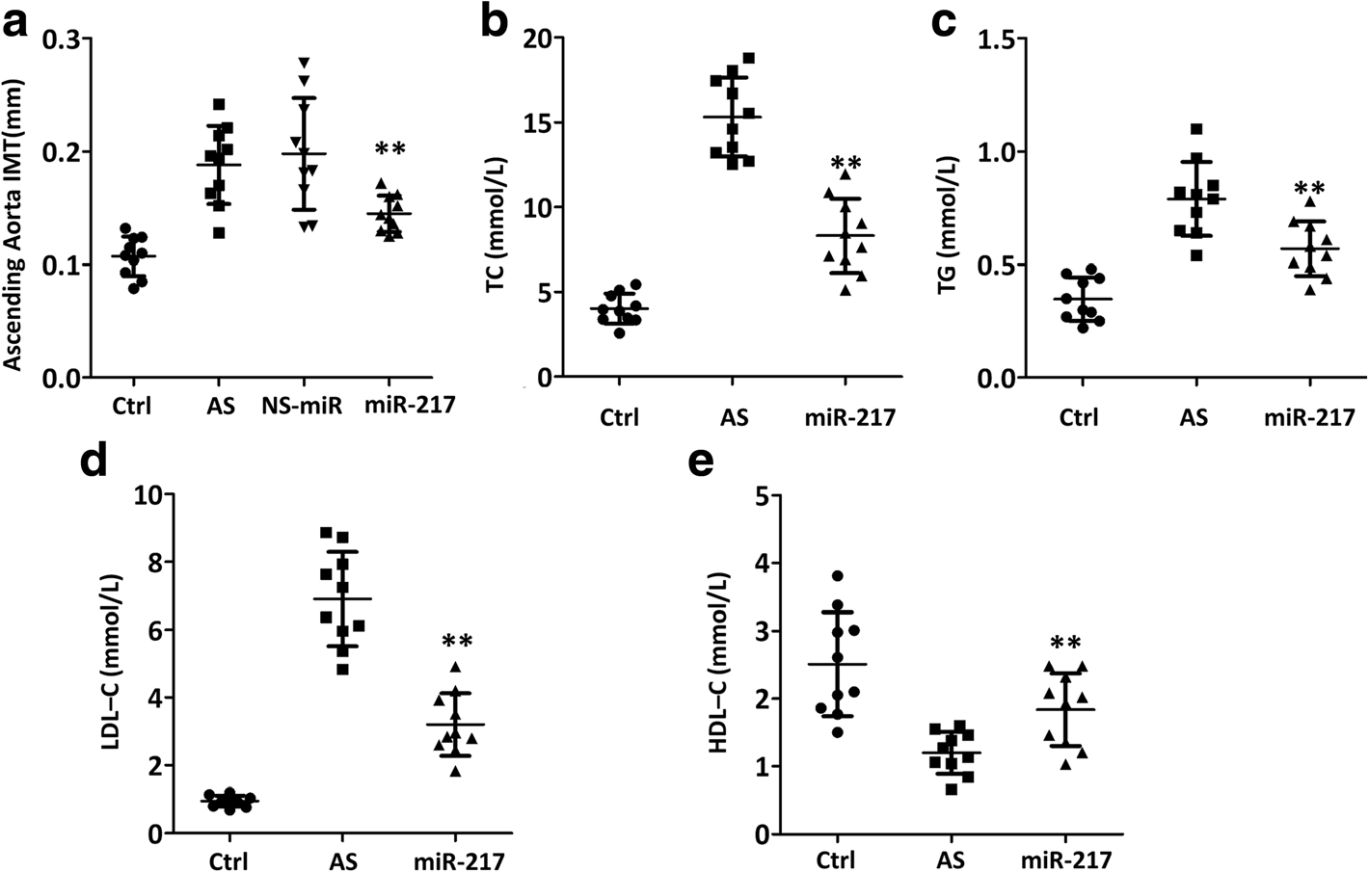
miR-217 mimic attenuated ascending aorta intima–media thickness (**a**) and serum lipid levels, including TC (**b**), TG (**c**), LDL-c (**d**) and HDL-c (**e**), in atherosclerotic models of ApoE^−/−^ mice. TC = total cholesterol; TG = triglycerides; LDL-C = low-density-lipoprotein cholesterol; HDL-C = high-density-lipoprotein cholesterol. *N* = 10 mice, **p < 0.01 vs. AS group

The presence and thickness of atherosclerotic plaque can be observed in the ApoE^−/−^ mice in the ascending aorta as seen by IMT, which indicated various degrees of atherosclerosis (Fig. 3a). miR-217 mimic attenuated IMT of ascending aorta when compared with AS group (Fig. 3a, *p* < 0.01), and NS-miR administration did not alter the IMT of ascending aorta, which indicated the specific function of miR-217 mimic. It was also demonstrated that miR-217 mimic could decrease the serum level of TC, TG, and LDL-C in ApoE^−/−^ mice (Fig. 3b–d, *p* < 0.01) and up-regulate the serum level of HDL-C (Fig. 3e, *p* < 0.01).

### miR-217 down-regulates the expression of inflammation and lipid metabolism-related genes in the aorta

**Fig. 4:**
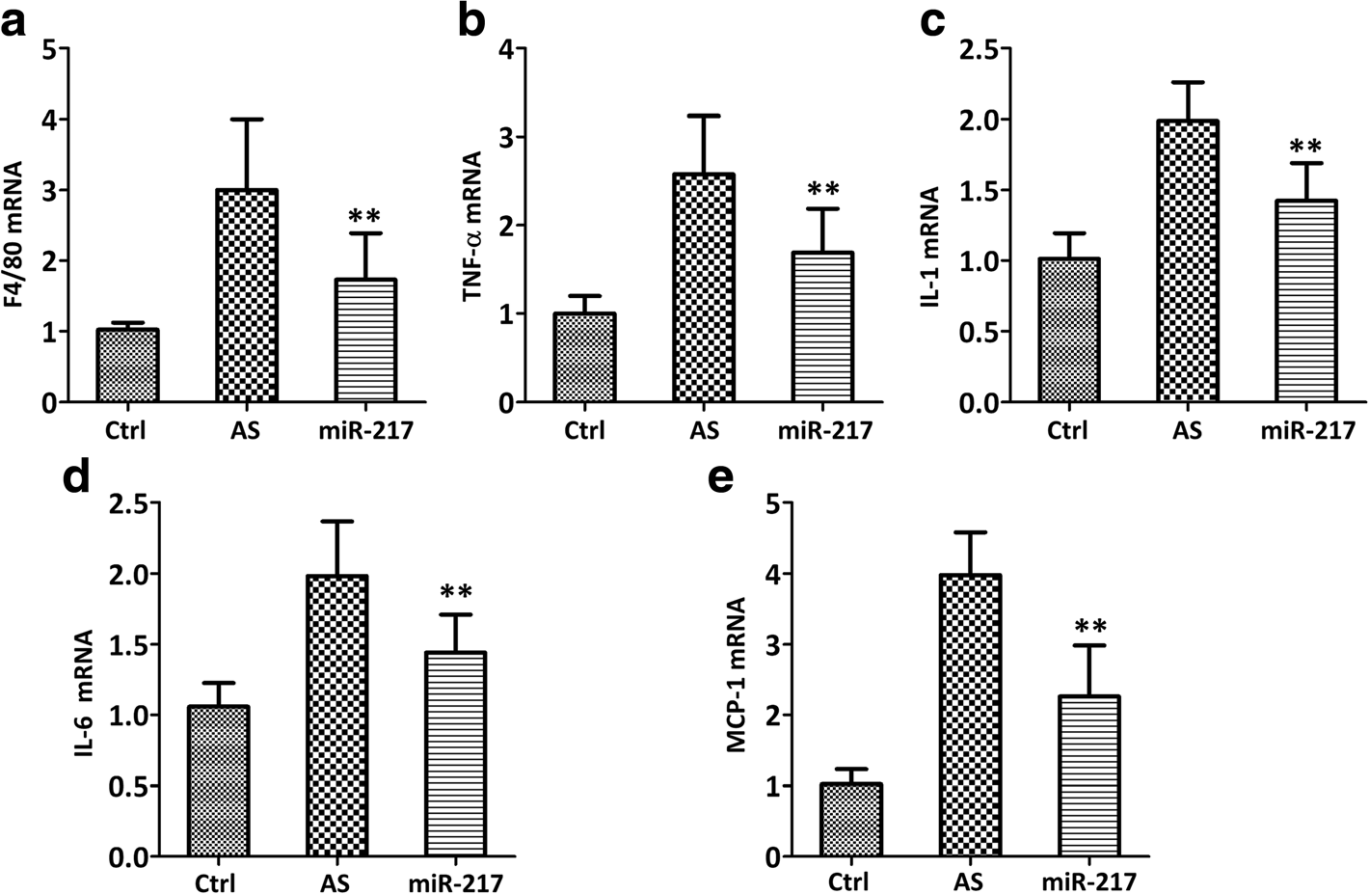
miR-217 mimic reduced the levels of inflammatory cytokines, as evidenced by the decreases of mRNA expressions of F4/80 (**a**), TNF-α (**b**), IL-1 (**c**), IL-6 (**d**) and MCP-1 (**e**), in atherosclerotic models of ApoE^−/−^ mice. qRT-PCR was used to test inflammatory cytokine genes in the aorta. *N* = 5 mice, **p < 0.01 vs. AS group

**Fig. 5:**
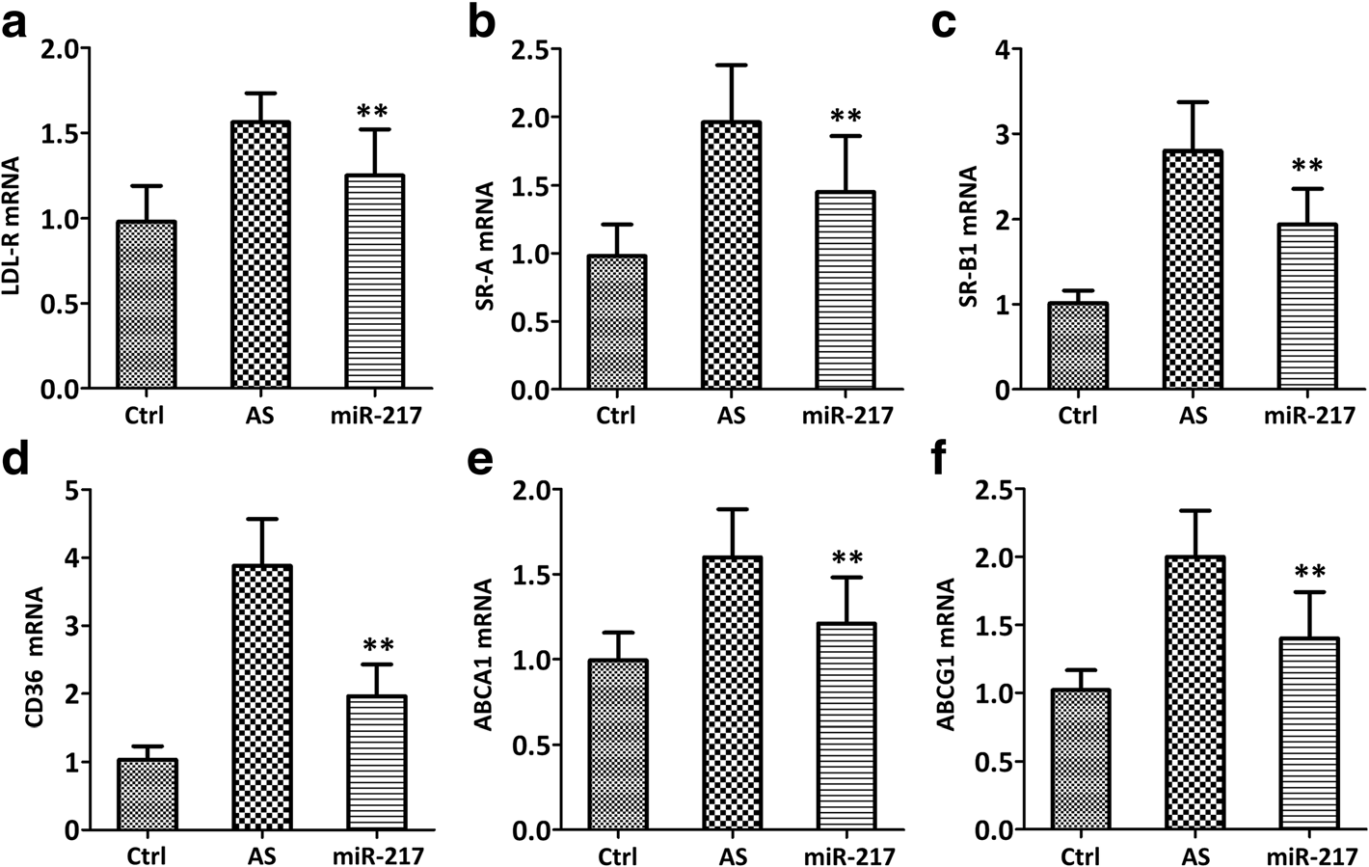
miR-217 mimic reduced the levels of lipid metabolism-related genes, including LDL-R (**a**), SR-A (**b**), SR-B1 (**c**), CD36 (**d**), ABCA1 (**e**) and ABCG1 (**f**), in atherosclerotic models of ApoE^−/−^ mice. qRT-PCR was used to test lipid metabolism-related genes in the aorta. *N* = 5 mice, **p < 0.01 vs. AS group

miR-217 mimic could down-regulate the expression of both the macrophage marker (F4/80) (Fig. 4a, *p* < 0.01) and inflammatory cytokine, such as tumor necrosis factor (TNF)-α (Fig. 4b, *p* < 0.01), interleukin (IL)-1(Fig. 4c, *p* < 0.01), IL-6 (Fig. 4d, *p* < 0.01), and monocyte chemoattractant protein (MCP)-1 (Fig. 4e, *p* < 0.01) as compared with the untreated AS model. It was also detected that lipid metabolism-related genes, such as LDL receptor (LDL-R) (Fig. 5a, *p* < 0.01), class A scavenger receptors (SR-A) (Fig. 5b, *p* < 0.01), scavenger receptor class B type I (SR-BI) (Fig. 5c, *p* < 0.01), CD36 (Fig. 5d, *p* < 0.01), ATP binding cassette subfamily A member 1 (ABCA1) (Fig. 5e, *p* < 0.01), and ATP binding cassette subfamily G member 1 (ABCG1) (Fig. 5f, *p* < 0.01) were significantly down-regulated in miR-217 mimic group when compared with AS group.

## Discussion

In this research, the expression level of miR-217 in the serum shows a correlation with the intima–media thickness of the ascending aorta measured by ultrasound bio-microscopy in ApoE^−/−^ mice, which is further testified to attenuate IMT and inhibit inflammatory factors secretion and lipid metabolism involved in atherosclerosis formation. This study focuses on the change of miR-217 in extracellular/circulating level and also emphasizes the potential beneficial effects of miR-217 in AS treatment.

Concomitant risk factors may contribute to the development of atherosclerosis. Atherosclerosis-prone ApoE^−/−^ mice may develop dyslipidemia with subsequent accumulation of cholesterol ester-enriched particles in the blood, which not only induces a chronic inflammatory reaction in the vessels but also promotes the development of atherosclerotic plaques [19]. Excess LDL-C deposit in the subendothelial space may lead to endothelial cells (ECs) activation and monocytes recruitment. Macrophages derived from monocytes could engulf the oxidized or denatured LDL via scavenger receptors, such as SR-A, SR-B, and CD36, and recruit further inflammatory cells (T-cells and B-cells) into the vascular intimal layer. In this context, after infiltrating the aorta, F4/80^+^ macrophages undergo foaming and lesions can develop from early fatty streaks to complex vulnerable plaques, which may contribute to the clinical consequences of myocardial infarction or stroke [20]. In this study, our data demonstrate that not only the marker of macrophages (F4/80) but also inflammation (TNF-α, IL-1, IL-6, and MCP-1) and lipid-related molecules (LDL-R, SR-A, SR-B1, and CD36) in the aorta are inhibited by miR-217 mimic administration. It is interesting to find that both ABCA1 and ABCG1 are down-regulated which are responsible for the major part of macrophage cholesterol efflux to serum or HDL in macrophage foam cells [21]. All of these indicate that other efficient pathways such as passive efflux may be also involved. Combined with decreased serum lipid levels and attenuated IMT after miR-217 mimic treatment, it can be deduced that miR-217 has the ability to inhibit the formation of plaque in atherosclerosis.

It is worth noting that statins (3-hydroxy-3-methylglutaryl coenzyme A reductase inhibitors) which can favorably alter plaque size, chemical composition, and inflammation and cholesterol metabolism, has significant clinical benefit during 11 years of follow-up clinical trials [22]. miR-217 might also be the key player in AS biology, for it can regulate both the inflammation and the cholesterol metabolism processes, which can profoundly affect plaque evolution. Whether miR-217 has such notable effect and further detailed mechanism need more research.

Ultrasound bio-microscopy enables evaluation of plaque size and stenosis in vivo, noninvasively, and in real-time [17, 23–25], which has been widely used to detect spontaneously formed plaques in the brachiocephalic [26, 27], thoracic arteries [24, 28], and the ascending aorta [23]. In this study, such technique is used to monitor the development of plaque of atherosclerosis and such technique will give the longitudinal study possible.

Our research indicates that UBM enables in vivo, noninvasively monitor of atherosclerotic plaque lesions. Serum miR-217 not only correlates with the IMT of the ascending aorta measured by UBM in ApoE^−/−^ mice but also attenuates the formation of plaque. Further research testifies that miR-217 can inhibit inflammation factors secretion and lipid deposition involved in atherosclerosis formation. All of these indicate that miR-217 might be a potential new treatment strategy.

## Conclusion

Although further detailed mechanism and serum cytokines levels need to be detected, our data indicate that miR-217 could be used to treat atherosclerosis.

## Abbreviations

ABCA1: ATP binding cassette subfamily A member 1
ABCG1: ATP binding cassette subfamily G member 1
IL-1: Interleukin
MCP-1: Monocyte chemoattractant protein
SR-A: Class A scavenger receptors
SR-BI: Scavenger receptor class B type I
TNF-α: Tumor necrosis factor
HDL-C: High-density lipoprotein cholesterol
IMT: Intima–media thickness
LDL-C: Low-density-lipoprotein cholesterol
miR-217: MicroRNA-217
TC: Total cholesterol
TG: Triglyceride
UBM: Ultrasound bio-microscopy
